# Treatment in patients’ best interests

**DOI:** 10.1080/17571472.2016.1225627

**Published:** 2016-09-20

**Authors:** Lindsay Gee

**Affiliations:** ^a^ Clinical Law, Capsticks Solicitors LLP, Leeds, UK

**Keywords:** Autonomy, incapacity, urgent treatment

## Abstract

A note on the current state of the law as regards balancing the autonomy, and personal values, of a mentally incapacitated patient against treatment plans with which they do not agree. The author summarises the outcomes of two medical treatment cases heard by the Court of Protection, with similar facts and different results, by way of adding to the advice to practitioners for their communications with patients and families in such situations.

## Why this matters to me

Because the two cases together give a clear picture of the importance of weighing up the wishes, feelings, beliefs and values of a mentally incapacitated patient when deciding whether to give them treatment to which they object; and illustrate that their autonomy may override the need to give them treatment even if this may be a matter of life and death.

## Key message

That you should not assume that the medical imperative of giving particular treatment, even life-saving treatment, to individuals will automatically outweigh their objections to it because they lack capacity to decide. Their exercise of autonomy, as an expression of their personality and a reflection of their lives, may result, in the last resort, in a Judge’s order that the treatment should not be given because it is, in the circumstances, not in the patient’s best interests. Wide consultation is always desirable to help you ‘put yourself in the shoes’ of the patient from when they were in full health; while devising advance decisions, and other records of patients’ wishes and values, while they still have capacity will make such dilemmas relatively easier.

## Treatment in patients’ best interests

In line with the tenets of the Mental Capacity Act and current medical ethics, one message here is that the widest possible consultation about patients’ wishes, feelings, beliefs and values should be carried out with their friends and families, taking into consideration what is known, or can be concluded, about their subjective quality of life. The medical duty of care extends to respecting the individual’s own wishes and values even in the face of incapacity and death, and with the aid of continuing dialogue between doctor and patient. This will sometimes require doctors to stand back and let nature take its course.

A second, related message is that everything possible should be done to become familiar with patients’ concerns while they still have capacity. For example by means of family conferences, and by encouraging people to make advance decisions (‘living wills’) or to grant a (preferably younger) relative or friend a Lasting Power of Attorney, with a view to their wishes being recorded so that they can be acted on, or respected, in the future.

But whenever there remains doubt or disagreement about the treatment proposal, the Court of Protection must be approached to arbitrate. In some cases a mentally incapacitated patient’s refusal may be upheld by a Judge even if the consequence is that the patient will die.

Take the example of a GP faced with a patient suffering from dementia, advanced cancer and kidney failure, who is vigorously resisting dialysis. The first step is to obtain, and document, an up-to-date assessment as to whether the patient has lost the capacity to decide on that treatment. If so, the Judge will review the evidence on the patient’s reasons for refusing dialysis, the family’s views, the management difficulties, the prognosis, and the perceived future quality of life, alongside the need to tackle the kidney failure, before reaching a decision, which will then determine what course of action will be followed.

So how does this work in practice? Consider this first scenario,[1] from July 2015. ‘Ms AB’, a woman in her late sixties, presented with an ulcerated leg, after a scalding injury to her foot, in which necrosis and osteomyelitis had developed. She suffered from poorly-controlled diabetes; and from severe depression, with delusional beliefs about the staff and about her own power to treat the wound, to the extent that she was incapable of understanding the danger she was in. She objected strongly to surgery: and it was recognised by everyone involved that it would have an adverse impact on her mental state and future quality of life. However, the only alternative to above-knee amputation was considered to be death, and in the near future. With evidence from several clinicians before him, and despite foreseeable future difficulties, the Judge was satisfied that it was in Ms AB’s best interests to undergo the surgery. This was then carried out.

Now consider this second scenario,[2] from September 2015. A 73-year-old patient, ‘Mr B’, had a half-century history of schizo-affective disorder, poorly-controlled diabetes and a chronic foot ulcer which had progressed to osteomyelitis. This presented a stark choice between below-knee amputation and dying. Post-operative life expectancy was estimated at 3 years.

Mr B was implacably opposed to the amputation. It conflicted with his religious beliefs. There was a consensus that he lacked capacity. The psychiatric and orthopaedic teams were convinced that it was in his best interests to perform the surgery. The Official Solicitor’s experts, a consultant vascular surgeon and a consultant psychiatrist, agreed.

The Judge met Mr B himself and observed that fierce independence of spirit was a core aspect of his personality. He then refused to declare that it was in Mr B’s best interests to undergo surgery without his consent. In *this* case autonomy trumped the preservation of life: just as it does for any patient who possesses capacity to refuse.

In the Judge’s view, the surgery would leave Mr B with a future for which he had little appetite, and an inability to carry on with activities he enjoyed or to live in his own home. His wishes were to be respected, notwithstanding the team’s desire to save his life. ‘There is a difference between fighting on someone’s behalf and just fighting them.’ This left the team with one course of action, palliative care.

A decision of this kind avoids discriminating against a disabled patient by treating them less favourably than someone without disabilities. Instead it is a matter of appraising ‘best interests’ in the context of what is most important to each particular patient.

## Governance

The patients are not named, but anonymised as required by the Court. No conflict of interest.

## Disclosure statement

No potential conflict of interest was reported by the author.
